# Indenopyrans – synthesis and photoluminescence properties

**DOI:** 10.3762/bjoc.12.81

**Published:** 2016-04-27

**Authors:** Andreea Petronela Diac, Ana-Maria Ţepeş, Albert Soran, Ion Grosu, Anamaria Terec, Jean Roncali, Elena Bogdan

**Affiliations:** 1 Babeş-Bolyai University, Center of Supramolecular Organic and Organometallic Chemistry (CSOOMC) Cluj-Napoca, 11 Arany Janos, 400028 Cluj-Napoca, Romania; 2 Group Linear Conjugated Systems, CNRS Moltech-Anjou, University of Angers, 2 Boulevard Lavoisier 49045 Angers, France

**Keywords:** indenopyran-3-ones, organic fluorophores, pyrones, solid-state emission spectra, solution emission spectra, UV–vis spectra

## Abstract

New indeno[1,2*-c*]pyran-3-ones bearing different substituents at the pyran moiety were synthesized and their photophysical properties were investigated. In solution all compounds were found to be blue emitters and the *trans* isomers exhibited significantly higher fluorescence quantum yields (relative to 9,10-diphenylanthracene) as compared to the corresponding *cis* isomers. The solid-state fluorescence spectra revealed an important red shift of λ_max_ due to intermolecular interactions in the lattice, along with an emission-band broadening, as compared to the solution fluorescence spectra.

## Introduction

Indenopyrans are functionalized oxygen-containing heterocycles consisting of fused indene and pyran units. Generally, oxygen-containing heteroaromatic compounds have been encountered as biologically active molecules and display exciting physico-chemical properties. Therefore, these compounds have found many applications in medicinal chemistry and material sciences. Besides, the annelated cyclic indenes containing N or O heteroatoms are the main structural frameworks of several natural alkaloids [1], while indenopyran derivatives were found to be valuable substrates for the synthesis of various compounds with biological activity [2–3]. Some indeno[1,2-*c*]isochromen-5(11*H*)-ones have been reported as key intermediates for the development of several topoisomerase I (Top1) anticancer agents [4–7]. A natural product exhibiting very promising antitumor activity is β-lapachone having a naphtho[1,2-*b*]pyrandione skeleton [8–9]. Also worth mentioning in this context are benzo[*b*]indeno-[2,1-*e*]pyran-10,11-diones, which stimulate the biosynthesis of erythropoietin and are used to treat anemia [10]. Another application includes the use of some amino-spiroindenopyran derivatives as selective sensors for thallium(I) ions in human urine [11].

Pyran-containing derivatives have been found to act as potential candidates for electroluminescent devices due to their fluorescent properties. Fluorescent dyes have lately attracted considerable interest owing to their wide range of applications in various fields. For example, naphthopyrans (e.g. 3,3-diphenyl-3*H*-naphtho[2,1-*b*]pyrans) are important photochromic compounds used for the fabrication of plastic lenses [12–13]. Their photochromic properties were improved through an extended conjugation through the fusion to an indeno group. Moreover, 2-alkyl-6-(4-(dimethylaminostyryl)-4*H*-pyran, 2,3-dihydro-1*H*-cyclopenta[3*a*,8*a*]indolin-5-enyl)-4*H*-pyran [14–17] and benzopyran (4-dicyanomethylenechromene) [18–19] have been employed as red doping agents for organic light emitting diodes (OLEDs). Furthermore, pyran derivatives decorated with electron-donating 6-(4-diethylamino)phenyl and electron-withdrawing cyano groups have been found to exhibit good solvatochromism properties and high quantum yields in the solid state why they are considered as important precursors for the fabrication of fluorescent materials [20].

Owing to their exciting photophysical properties many dicyanomethylene pyran-containing chromophores are valuable candidates for the fabrication of OLEDs [21–22], dye lasers [23], sensors [24], dye-sensitized solar cells, fluorescent probes [25] or logic gates [26]. Recently [27], 6-CF_3_-2*H*-pyran-2-ones have been reported as potential building blocks for the preparation of novel trifluoromethylated heterocycles, such as indone and carbostyril derivatives.

Based on their important biological functions in nature and the synthetic potential of α-pyrones for the construction of a variety of fluorescent heteroarenes, we decided to synthesize and study the optoelectronic properties of some indeno-fused pyran-3-ones and the results are reported herein.

## Results and Discussion

**Scheme 1:**
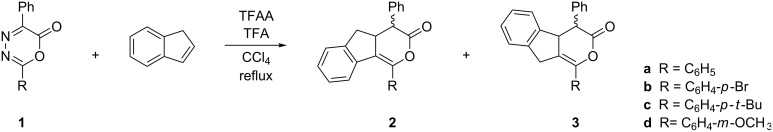
Synthesis of dihydroindeno[1,2*-c*]pyran-3-ones **2** and **3**.

For a long time, the Diels–Alder reaction of simple heterosubstituted 1,3-dienes has been considered a standard procedure to prepare highly functionalized ring systems [28]. Taking advantage of diazalactones to act as the dienophile component in inverse-electron demand Diels–Alder reactions, a series of new dihydroindeno[1,2-*c*]pyran derivatives was synthesized. Thus, the addition of freshly distilled indene as a diene component to oxadiazinones **1** as the dienophiles [29] under acidic conditions (TFA, TFAA) led to the formation of dihydroindeno[1,2*-c*]pyran-3-ones **2** and **3** as a mixture of isomers (Scheme 1). Additionally, the corresponding phenyl-substituted compounds **2a** and **3a** (previously reported [30]) were synthesized to investigate the effect of an aryl substituent in position 1 on the photophysical properties of the compounds. The selective formation of type **3** isomers has been reported earlier [31–32]. They are accessible through a cycloaddition reaction of indene and methyl 6-oxo-5-phenyl-6*H*-1,3,4-oxadiazine-2-carboxylate followed by the treatment with bromine (or hydrogen chloride), thus yielding the corresponding halogenated lactones having the 4a,9a-fused skeleton and an ester group at position 1.

**Figure 1:**
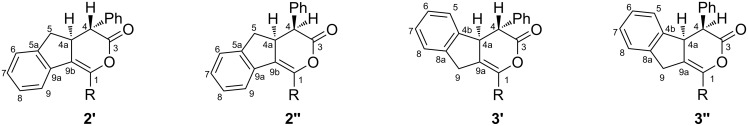
Possible isomers of dihydroindeno[1,2*-c*]pyran-3-ones **2** and **3**.

The cycloaddition reaction proceeded with moderate regio- and stereoselectivity. As reported in the literature, the inverse-electron demand Diels–Alder cycloaddition of unsymmetrical olefins and oxadiazinones [33–35] may lead to two possible regioisomers: isomer **2** with the benzene ring fused at C5a–C9a and its regioisomer **3** with the benzene ring fused at C4b–C8a (Figure 1).

Additionally, due to the new stereocenters created at positions 4 and 4a, each regioisomer could be expressed as a set of diastereoisomers. Thus, the isomers denoted **2'** and **3'** exhibit the phenyl group at position 4 and the indeno fragment at position 4a in a *trans* configuration, while the isomers **2''** and **3''** bear the two aforementioned groups in a *cis* configuration (Figure 1).

In most cases, regioisomer **2** predominates and occasionally, formation of derivative **3** has not even been observed (e.g., **d** derivatives). Moreover, the nature of the substituent influences the regio- and diastereoisomeric ratio (Table 1).

**Table 1:** Ratio of regio- and diastereoisomers **2** and **3**.^a^

	Ratio			
Compound	**2'**	**2''**	**3'**	**3''**

**a** R = C_6_H_5_	1	–	–	0.2
**b** R = C_6_H_4_-*p*-Br	1	1	–	0.25
**c** R = C_6_H_4_-*p*-*t*-Bu	1	1	0.2	–
**d** R = C_6_H_4_-*m*-OCH_3_	1.2	1	–	–

^a^Reaction conditions: TFAA (1.5 equiv), TFA (1.55 equiv), indene (6 equiv), CCl_4_, reflux, 9 d.

The diastereoisomers exhibit very close *R*_f_ values which makes their separation by conventional chromatography quite difficult. Nevertheless, successful separation of diastereoisomers **2'** and **2''** as individual isomers has been achieved for all compounds.

**Figure 2:**
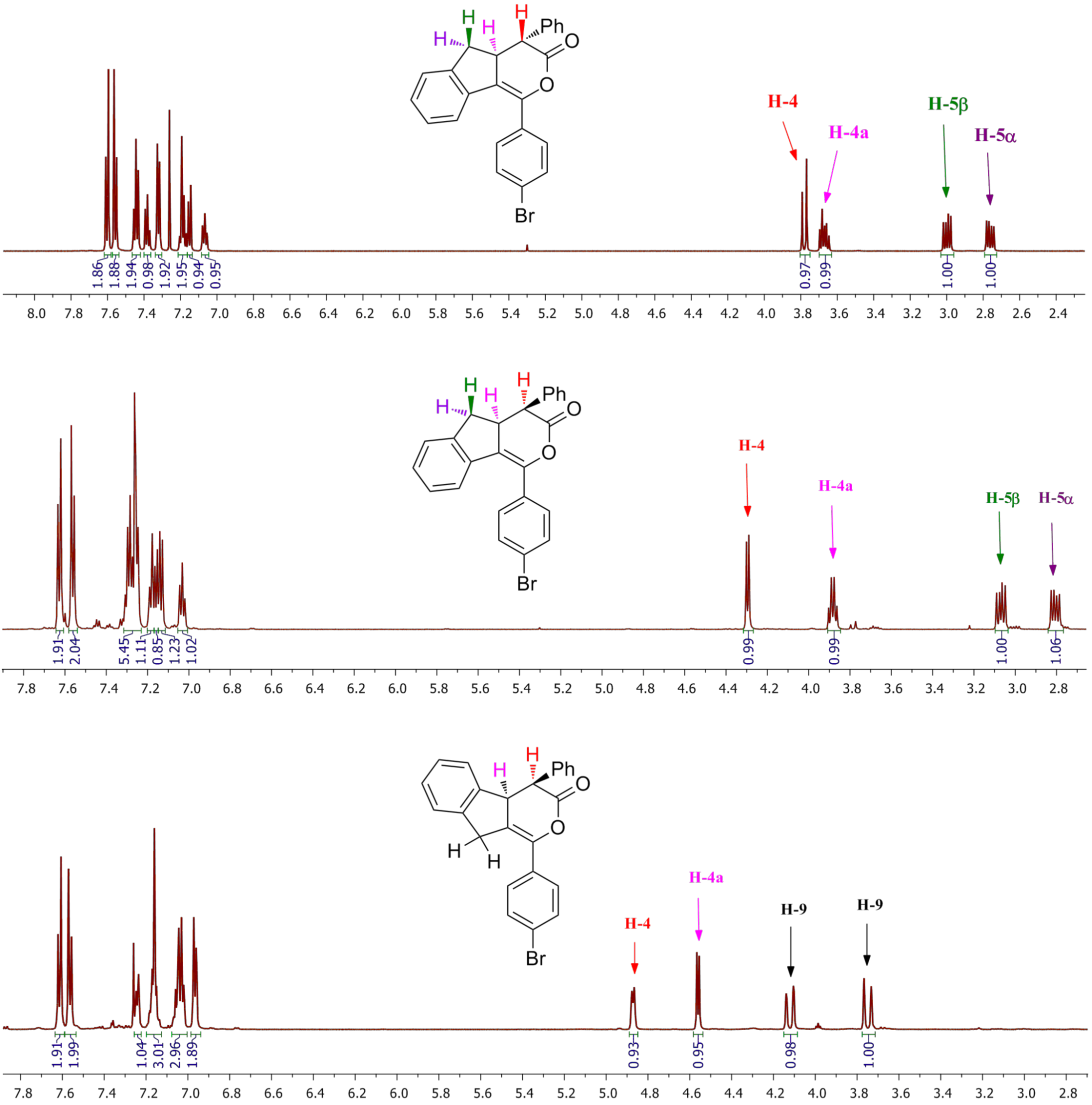
^1^H NMR spectra (600 MHz, CDCl_3_) of isomers **2'b** (top), **2''b** (middle) and **3''b** (bottom).

The configuration of the diastereoisomers was assigned based on ^1^H NMR spectroscopic measurements (Figure 2). Thus, for isomer **2'b**, the observed large coupling constant of 14.3 Hz indicates the *trans* orientation of H-4 relative to H-4a, while in case of isomer **2''b** the proton H-4 appears as a more deshielded doublet with a 7.1 Hz coupling constant, which is typical for the *cis* configuration. The *cis* configuration was also assigned for diastereoisomer **3''b** which displays one doublet at 4.87 ppm for the hydrogen atom at position 4 with a coupling constant of 6.7 Hz.

Generally, the expected structures for all compounds were fully confirmed by the characteristic signals in the ^1^H NMR spectra (Figure 2). The protons at positions 5 and 9 of compounds **2** or **3** exhibit large coupling constants, typically observed for geminal protons. Noteworthy, in case of isomer **3''b**, the specific methylene protons connected to the enol-lactone moiety display more deshielded signals than those of isomer **2''b** (Figure 2).

**Figure 3:**
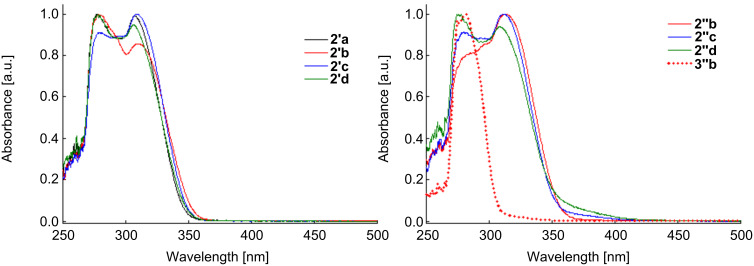
Normalized absorption spectra of dihydroindenopyrones **2'a**–**d**, **2''b**–**d** and **3''b**, recorded in acetonitrile (298 K).

Once the 1,4-disubstituted indenopyrone derivatives were synthesized and their structures confirmed by spectroscopical methods, we further investigated their photophysical properties. No significant differences could be observed in the shape of the electronic bands in the absorption spectra of **2a**–**d** recorded in acetonitrile (Figure 3) and methylene chloride (Figure S1, Supporting Information File 1). The spectra exhibit intense broad absorption bands between 275–282 nm and 307–317 nm, respectively, corresponding to π–π^*^ and n–π^*^ transitions. As a general remark, when compared to the reference compound **2a**, the maxima of absorption bands for **2b**–**d** are slightly red-shifted due to the substituents in position 1: C_6_H_4_-*m*-OCH_3_, C_6_H_4_-*p*-*t*-Bu and C_6_H_4_-*p*-Br, respectively (Table 2). The use of methylene chloride or acetonitrile as solvents led to a similar trend of the electronic spectra depending on the nature of the substituents (Table 2, Figure 3, Figure S1 in Supporting Information File 1). However, a slight bathochromic shift could be observed when methylene chloride was used in contrast to a hypsochromic shift in the more polar solvent acetonitrile.

**Table 2:** Absorption data for derivatives **2a**–**d** and **3''b** measured in acetonitrile and methylene chloride at rt.

	Acetonitrile			Methylene chloride		
	λ_abs_ (nm)	conc. (M)	log ε	λ_abs_ (nm)	conc. (M)	log ε

**2'a**	278; 307	7.3E−05	3.95; 3.95	280; 309	7.45E−05	3.97; 3.99
**2'b**	280; 310	5.75E−05	4.00; 3.94	280; 315	6.47E−05	3.89; 3.83
**2''b**	280; 295 (sh); 313	7.81E−05	3.80; 3.83; 3.90	282; 295 (sh); 317	7.79E−05	3.77; 3.82; 3.92
**3''b**	275; 282	7.38E−05	3.97; 3.98	275; 282	6.85E−05	4.05; 4.06
**2'c**	278; 308	8.92E−05	3.83; 3.87	282; 294 (sh); 312	6.34E−05	3.90; 3.91; 3.97
**2''c**	280; 312	7.73E−05	3.70; 3.74	280; 294 (sh); 314	7.00E−05	3.79; 3.80; 3.86
**2'd**	276; 307	6.43E−05	3.88; 3.86	280; 309	6.87E−05	3.84; 3.87
**2''d**	276; 309	7.6E−05	3.70; 3.68	280; 311	6.98E−05	3.71; 3.74

The analysis of the absorption spectra revealed some differences between the regioisomers **2''b** and **3''b** and diastereoisomers **2'** and **2''**, as well (Figure 3, Figure S1, Supporting Information File 1). Thus, a red shift of 35 nm was observed when comparing the longest wavelength for **2''b** (λ_max_ = 317 nm) with that of its regioisomer **3''b** (λ_max_ = 282 nm). This behavior could be explained by an extended electron delocalization in the conjugated system of isomer **2''b**. From the spectra obtained from the diastereoisomers **2'** and **2''**, one can observe a small shift to higher wavelengths in case of the *cis* isomers **2''**. Moreover, the spectrum of the *trans* isomer **2'b** displays the most intense absorption band at a lower wavelength (280 nm) in contrast to the *cis* isomers **2''b** (313 nm, in acetonitrile).

**Figure 4:**
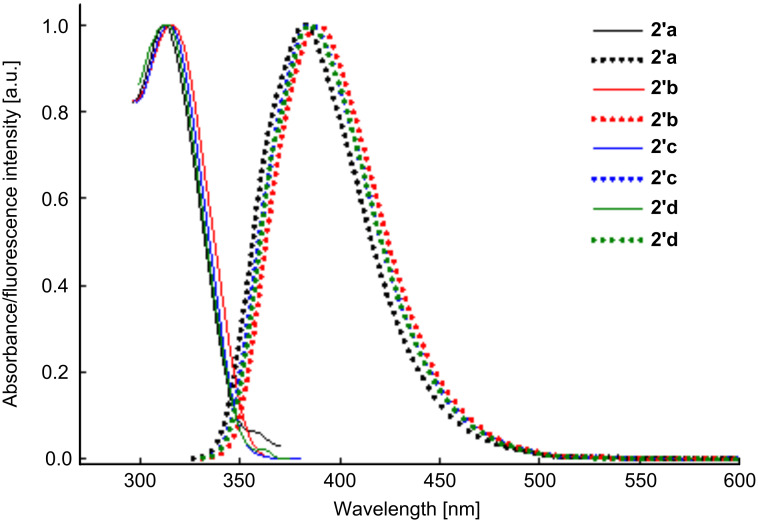
Normalized UV–vis (left) spectra at excitation wavelengths and fluorescence (right) spectra of dihydroindenopyrones **2'a**–**d**.

All derivatives **2a**–**d** showed an intense emission in acetonitrile in the range 380–393 nm (Figure 4, Table 3), while for the *cis* isomer **3''b** a weaker emission was observed. This may be explained by a less extended conjugation and an intersystem-crossing process that occurs when an electron is transferred from a singlet energy level into a triplet energy level [36].

**Table 3:** Absorption and emission data for dihydroindenopyrone derivatives **2'a**–**d**, **2''b**–**d** and **3''b**.

Compound	Excitation λ [nm]^a^	Emission λ [nm]^a^	Stokes shift [nm] / [ cm^−1^]	Emission λ [nm]^b^	Ф^c^

**2'a**	313	382	69 / 5771	423	7.2%
**2'b**	316	389	73 / 5939	445	18.1%
**2''b**	320	393	73 / 5805	432	5.4%
**3''b**	317	390	73 / 5905	–	0.3%
**2'c**	315	386	71 / 5839	–	12.6%
**2''c**	288	338; 356; 370	68 / 6632	475	7.2%
**2'd**	313	385	72 / 5975	461	10.8%
**2''d**	321	389	68 / 5446	491	3.6%

^a^Recorded in acetonitrile. ^b^In the solid state. ^c^Determined in acetonitrile with 9,10-diphenylanthracene as reference (Ф = 90%) [37].

The derivatives **2a**–**d** are blue emitters, displaying maximum-emission bands at around 370–393 nm. Remarkably, the fluorescence quantum yields calculated relative to 9,10-diphenylanthracene are significantly higher in the case of the *trans* isomers **2'** as compared to the corresponding *cis* isomers **2''** (Table 3).

**Figure 5:**
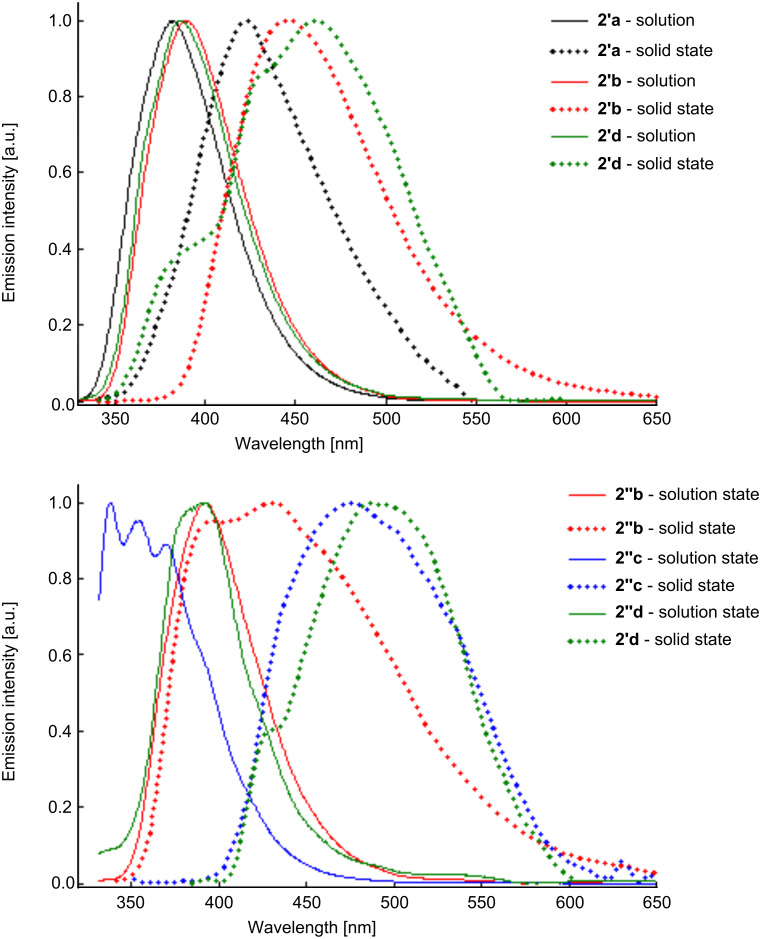
Normalized solid-state and solution (acetonitrile) fluorescence spectra of diastereoisomers **2a**–**d**.

The solid-state photoluminescence properties of the dihydroindenopyrones **2'a**–**c** and **2''a**–**d** were also investigated. These derivatives exhibit emission maxima in the range 423–491 nm when excited at 310 nm (Figure 5, Table 3).

Comparing the solution (acetonitrile) and solid-state fluorescence spectra of both diastereoisomers (Figure 5), one can observe a bathochromic shift of λ_max_ in the solid state and a broadening of the emission band because of intermolecular interactions.

**Scheme 2:**
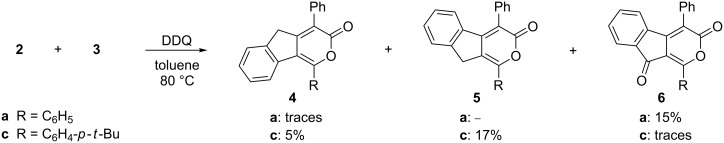
Synthesis of α-pyrones **4**–**6**.

Next, in order to obtain photoluminescent compounds with higher performance, the dehydrogenation reaction of enol-lactones **2** and **3** was performed. Adapted from a procedure previously described [38], the oxidation of the isomeric mixture **2'a**/**3''a** and **2'c**/**3'c**, respectively, in the presence of 2,3-dichloro-5,6-dicyano-1,4-benzoquinone (DDQ), led to indeno-α-pyrones **4**–**6** (Scheme 2), in not very satisfying yields.

Investigations carried out for the dehydrogenation reaction of the isomeric mixture **2'a**/**3''a** (Scheme 2) revealed the formation of the α-pyrone **6a** (15% yield), which was the oxidation product of isomer **3''a**. In the same time, most of the isomer **2'a** was recovered and traces of derivative **4a** could only be detected in the NMR spectrum.

Using the same procedure, the enol-lactone isomeric mixture **2'c**/**3'c** was subjected to the dehydrogenation reaction yielding the regioisomers **4c** and **5c** (Scheme 2). The ^1^H NMR spectrum revealed the formation of the fully oxidized derivative **6c** in small amounts that could not be isolated. However, silica gel column chromatography of the reaction mixture allowed the recovery of the starting enol-lactone derivative **2'c** in 50% yield.

The ^1^H NMR spectra of α-pyrones **4c** and **5c** (Figure S4, Supporting Information File 1) exhibit characteristic signals for the aliphatic protons: one singlet at 4.19 ppm for derivative **5c** and one signal at 3.97 ppm for compound **4c**, respectively.

Next, the photoluminescence properties of the isolated indenopyrones **4c**, **5c** and **6a** were investigated. The UV–vis spectra (Figure S5, Supporting Information File 1) recorded in methylene chloride displayed two absorption maxima assigned to π–π* and n–π* transitions, respectively. All derivatives showed very weak emission in the range 380–550 nm (Figure S5, Supporting Information File 1) when excited at 370 nm, while the calculated quantum yields relative to 9,10-diphenylanthracene were quite low (<1%).

**Figure 6:**
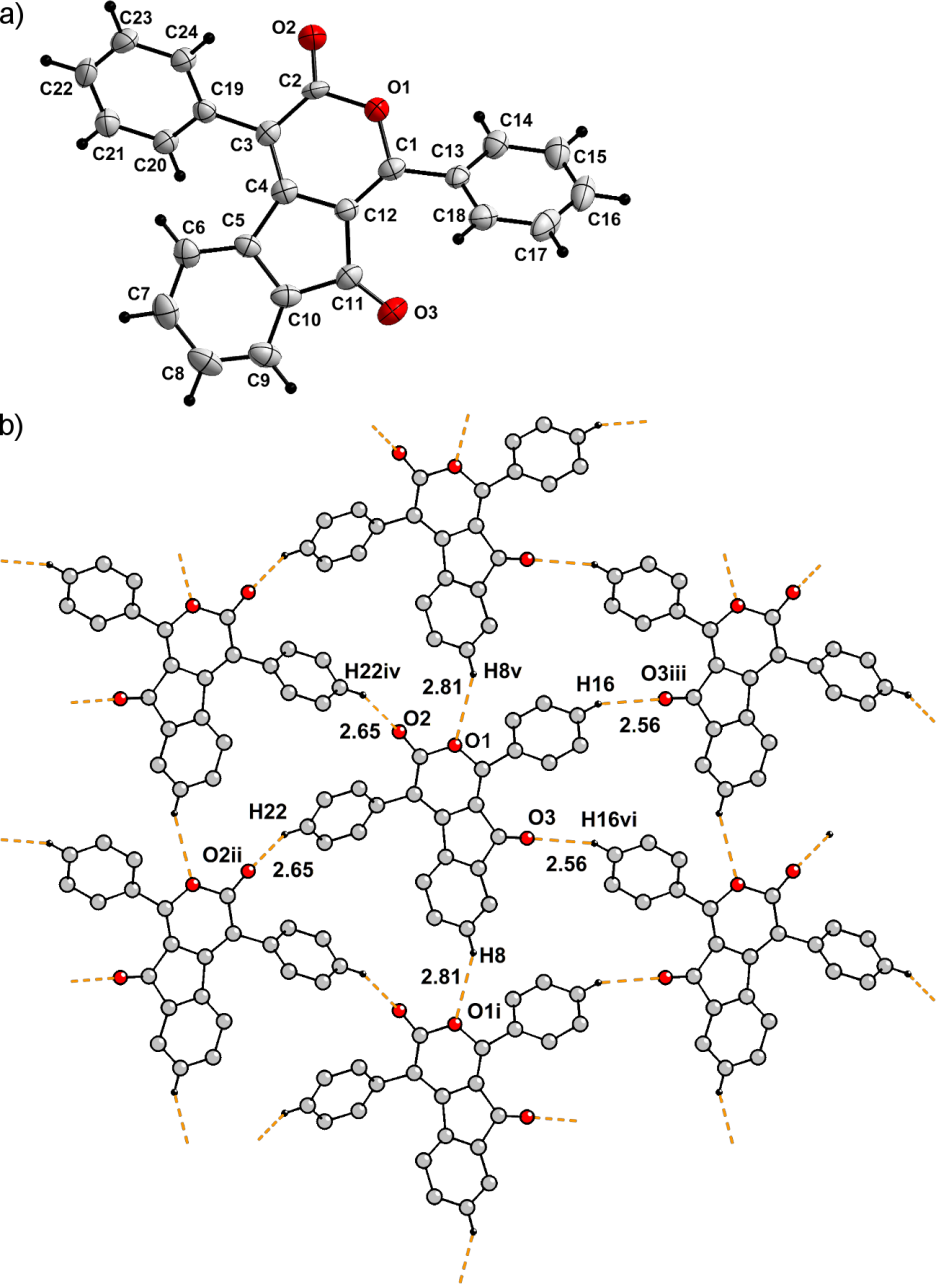
a) View of the asymmetric unit in the crystal of **6a**, shown with 40% probability ellipsoids. b) View along the *a*-axis in the crystal of **6a** showing a layer of molecules connected through weak C–H···O bonds (hydrogen atoms not involved in these weak bonds are omitted for clarity).

In the crystals of α-pyrone **6a**, the asymmetric unit consists of a single molecule. The two phenyl rings attached to the pyran-3-one moiety are disposed in a staggered conformation. The angle between the planes defined by the two phenyl rings is 85.8(1)° and the angle between the non-intersecting C1–C13 and the C3–C19 bond vectors is 12.9(3)°. Both phenyl rings are rotated with respect to the best plane defined through the pyran-3-one moiety (C1–O1–C(2–4)–C12), with angles of 35.8(2)° for the (C13–18) ring and 51.1(1)° for the (C19–24) ring, respectively (Figure 6a).

Several weak, intermolecular C–H···O hydrogen bonds could be identified in the crystal packing (Table S1, Supporting Information File 1). Although the H···O distances are close to or greater than the van der Waals limit of 2.60 Å [39], they are still within a range statistically observed for other compounds [40–44]. These C–H···O interactions lead to a first layer of molecules (Figure 6b) with the contribution of O1····H8, O2····H22 and O3····H16 bonds. Subsequently, these layers are connected through the weak O2···H15 and O2···H17 interactions.

Further edge-to-face H···π [44] and π–π interactions [45–46] (Table 4) between molecules from neighboring layers contribute to the three-dimensional crystal packing (Figure S6 in Supporting Information File 1).

**Table 4:** Intermolecular H···π and π–π interactions in the crystal of **6a**.

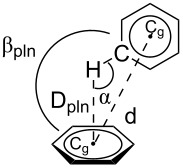			

C–H···π interactions			

C–H···pln	D_pln_ (Å)	α (°)	β_pln_ (°)
C20–H20···C(13–18)^a^	2.93	116	85.8
C18–H18···C(19–24)^b^	2.95	109	78.7
C14–H14···C(5–10)^a^	2.79	143	39.5

π···π interactions			

pln···pln	d (Å)		β_pln_ (°)
C(4–12)···C(4–12)^a^	3.52		0 within s.d.
C(4–12)···C(4–12)^b^	3.56		0 within s.d.

^a^Symmetry equivalent atoms are given by −x, 1−y, −z. ^b^Symmetry equivalent atoms are given by (ii) 1−x, 1−y, −z.

## Conclusion

Several indenopyrone derivatives were synthesized and characterized by NMR, UV–vis and fluorescence spectroscopy as well as mass spectrometry. Column chromatography of the crude product furnished the diastereoisomers as pure samples. The electronic spectra display two intense absorption bands ranging between 275–282 nm and 307–317 nm, while their photoluminescence spectra revealed that all compounds are blue emitters.

The calculated fluorescence quantum yield relative to 9,10-diphenylanthracene is significantly higher for the *trans* isomers when compared to the corresponding *cis* isomers. The solid-state fluorescence investigations revealed in the case of the regioisomers **2** a bathochromic shift together with an emission-band broadening in contrast to their emission spectra recorded in solution. The supramolecular arrangement in the solid state of indenopyrone **6a** is ensured by a number of non-covalent interactions such as intermolecular H-bonding, C–H···π and π···π contacts.

## Supporting Information

File 1 Experimental.
