# Supplementary material for: Indenopyrans – synthesis and photoluminescence properties
Source: Beilstein J Org Chem. 2016 Apr 27;12:825–34. doi: 10.3762/bjoc.12.81 (PMC4901902; doi:10.3762/bjoc.12.81)
Supplement: File 1 — Experimental. [file Beilstein_J_Org_Chem-12-825-s001.pdf]

Supporting Information

for

Indenopyrans – synthesis and photoluminescence properties

Andreea Petronela Diac[1], Ana-Maria Ţepeş[1], Albert Soran[1], Ion Grosu[1], Anamaria Terec[1], Jean Roncali[2], Elena Bogdan*[1]

Address: [1]Babeş-Bolyai University, Center of Supramolecular Organic and Organometallic Chemistry (CSOOMC) Cluj-Napoca, 11 Arany Janos, 400028 Cluj-Napoca, Romania and [2]Group Linear Conjugated Systems, CNRS Moltech-Anjou, University of Angers, 2 Boulevard Lavoisier 49045 Angers, France

Email: Elena Bogdan - ebogdan@chem.ubbcluj.ro
*Corresponding author

Dedicated to Professor Manfred Christl on the occasion of his 75th anniversary.

Experimental

General:

^{1}H and ^{13}C NMR spectra were recorded in $CDCl_3$ at room temperature on Bruker Avance 400 and 600 spectrometers (δ in ppm, *J* in Hz) at ^{1}H operating frequencies of 400 MHz (100 MHz for ^{13}C) and 600 MHz (150 MHz for ^{13}C), respectively. Spectra were referenced using the residual solvent signal as internal standard. HRMS in APCI mode ionization were recorded with an LTQ XL ThermoScientific mass spectrometer. UV–vis absorption spectra were carried out with a Cecil 9500 spectrophotometer using quartz cuvettes (1 cm). Fluorescence spectra were taken on a JASCO FP-8300 spectrofluorometer using glass cuvettes (1 cm). Melting points were measured with a Kleinfeld melting point apparatus and are uncorrected. Thin layer chromatography was performed on silica gel 60 chromatographic plates F_{254} and column chromatography was performed on silica gel. Solvents were dried and distilled under argon using standard procedures prior to use. Chemicals of commercial grade were used without further purification. Indene was freshly distilled prior to use. The supplementary crystallographic data for compound **6a** have been deposited at the Cambridge Crystallographic Data Centre, deposition number CCDC 1450552.

General procedure for the synthesis of dihydroindenopyrones 2 and 3:

TFAA (5 mmol, 0.7 ml), 5.25 mmol (0.4 ml) TFA and 30 mmol (3.5 ml) freshly distilled indene were added successively to a suspension containing 5 mmol of the corresponding oxadiazinone **1** in anhydrous tetrachloromethane (80 ml) under inert atmosphere. After the addition, the reaction mixture was refluxed under inert atmosphere while the suspension became a clear yellow solution. The color of the solution changed from yellow to red-brown and the reaction was monitored by TLC. After

5 days at reflux (pH solution changed from 1 to 4), additional TFAA (2.5 mmol, 0.35 ml) and 2.5 mmol (0.2 ml) TFA were added and the reaction was refluxed for 4 more days. After 9 days of reflux (pH 4) the solvent was removed in vacuum and the obtained brown residue was purified by column chromatography on silica gel to afford a white or yellow solid.

(4*S*,4a*R*)-1,4-Diphenyl-4a,5-dihydroindeno[1,2-*c*]pyran-3(4*H*)-one (2'a)

Chemical Formula: $C_{24}H_{18}O_2$; Molecular Weight: 338.40. White solid; yield 17%; mp. 151-152°C; R_f = 0.34 (pentane/methyl *tert*-butyl ether = 8/1). **^{1}H NMR** (400 MHz, $CDCl_3$) δ 2.77 (dd, 1H, $J_{5\alpha,5\beta}$ = 16.1 Hz, $J_{5\alpha,4a}$ = 7.6 Hz, H-5*α*), 3.00 (dd, 1H, $J_{5\beta,5\alpha}$ = 16.1 Hz, $J_{5\beta,4a}$ = 8.2 Hz, H-5*β*), 3.70 (m from overlapped ddd, 1H, H-4a), 3.80 (d, 1H, $J_{4,4a}$ = 14.2 Hz, H-4), 7.04 (m, 1H, H-7 or H-8), 7.13–7.20 (overlapped peaks, 3H, H-6, H-8 or H-7, H-9), 7.33 (m, 2H, *m'*-H, 1-C_6H_5), 7.38 (m, 1H, *p'*-H, 1-C_6H_5), 7.43–7.47 (overlapped peaks, 5H, 4-C_6H_5), 7.67 (m, 2H, *o'*-H, 1-C_6H_5) ppm. **^{13}C NMR** (100 MHz, $CDCl_3$) δ 37.0; 44.0; 52.4; 122.9; 124.1; 125.3; 127.0; 128.1; 128.4; 128.7; 128.8; 129.0; 129.1; 129.8; 132.9; 135.9; 136.5; 143.7; 145.7; 169.7 ppm. **UV-Vis** (CH_2Cl_2) λ_{max} (log ε) [nm]: 280 (3.97), 309 (3.99); (acetonitrile) λ_{max} (log ε) [nm]: 278 (3.95), 307 (3.95). **HRMS** (APCI+): calcd. for $C_{24}H_{19}O_2$ $[M+H]^+$: 339.1380; found: 339.1377; calcd. for $C_{23}H_{17}$ $[M-COOH]^+$: 293.1325; found: 293.1324.

(4*R*,4a*S*)-1,4-Diphenyl-4,4a-dihydroindeno[2,1-*c*]pyran-3(9*H*)-one (3''a)

Chemical Formula: $C_{24}H_{18}O_2$; Molecular Weight: 338.40. White solid; yield 4%, mp. 145-147°C; R_f = 0.25 (pentane/methyl *tert*-butyl ether = 8/1). **^{1}H NMR** (600 MHz, $CDCl_3$) δ 3.71 (d, 1H, $J_{9,9}$ = 17.4 Hz, 1H at position 9), 3.80 (d, 1H, $J_{9,9}$ = 17.4 Hz, 1H at position 9), 4.05 (d, 1H, $J_{4a,4}$ = 3.6 Hz, H-4a), 4.95 (d, 1H, $J_{4,4a}$ = 3.6 Hz, H-4), 7.21–7.36 (overlapped peaks, 9H, H-5, H-6, H-7, H-8, 4-C_6H_5), 7.50(m, 2H, *m'*-H (1-C_6H_5)), 7.62 (pseuso t from dd, 1H, *p'*-H (1-C_6H_5)), 8.08 (d, 2H, *J* = 7.8 Hz, *o'*-H (1-C_6H_5)) ppm. **^{13}C NMR** (150 MHz, $CDCl_3$) δ 45.4; 54.0; 57.2; 96.8; 124.8; 125.4; 127.8; 128.0; 128.6; 128.8; 128.9; 129.3; 130.3; 133.8; 134.0; 136.6; 138.2; 142.1; 175.7; 197.0 ppm.

(4*S*,4a*R*)-1-(4-Bromophenyl)-4-phenyl-4a,5-dihydroindeno[1,2-*c*]pyran-3(4*H*)-one (2'b)

Chemical Formula: $C_{24}H_{17}BrO_2$; Molecular Weight: 417.29. White solid; yield 24%; mp. 174–176°C; R_f = 0.30 (pentane/methyl *tert*-butyl ether = 8/1). **^{1}H NMR** (600 MHz, $CDCl_3$) δ 2.76 (dd, 1H, $J_{5\alpha,5\beta}$ = 16.1 Hz, $J_{5\alpha,4a}$ = 7.7 Hz, H-5*α*), 3.00 (dd, 1H, $J_{5\beta,5\alpha}$ = 16.1 Hz, $J_{5\beta,4a}$ = 8.4 Hz, H-5*β*), 3.67 (m from overlapped ddd, 1H, H-4*a*), 3.78 (d, 1H, $J_{4,4a}$ = 14.3 Hz, H-4), 7.04–7.20 (overlapped peaks, 4H, H-6, H-7,H-8, H-9), 7.31–7.47 (overlapped peaks, 5H, 4-C_6H_5), 7.56, 7.60 (2 x d, 4H, *J* = 8.5 Hz, 1-C_6H_4) ppm. **^{13}C NMR** (150 MHz, $CDCl_3$) δ 37.0; 44.2; 52.2; 122.8; 123.9; 124.7; 125.4; 127.1; 128.1; 128.7; 129.0; 129.1; 130.3; 132.0; 135.6; 136.1; 141.7; 142.3; 145.8; 169.5 ppm. **UV-Vis** (CH_2Cl_2) λ_{max} (log ε) [nm]: 280 (3.89), 315 (3.83); (acetonitrile) λ_{max} (log ε) [nm]: 280 (4.00), 310 (3.94). **HRMS** (APCI+): calcd. for $C_{24}H_{18}BrO_2$ $[M+H]^+$: 417.0485; found: 417.0422; calcd. for $C_{23}H_{16}Br$ $[M-COOH]^+$: 371.0430; found: 371.0384.

(4*R*,4a*R*)-1-(4-Bromophenyl)-4-phenyl-4a,5-dihydroindeno[1,2-*c*]pyran-3(4*H*)-one (2''b)

White solid; yield 23%; mp. 199–201°C; R_f = 0.2 (pentane/methyl *tert*-butyl ether = 8/1). **^{1}H NMR** (600 MHz, $CDCl_3$) δ 2.81 (dd, 1H, $J_{5\alpha,5\beta}$ = 16.0 Hz, $J_{5\alpha,4a}$ = 8.0 Hz, H-5*α*), 3.07 (dd, 1H, $J_{5\beta,5\alpha}$ = 16.0 Hz, $J_{5\beta,4a}$ = 9.0 Hz, H-5*β*), 3.88 (m from overlapped ddd, 1H, H-4a), 4.30 (d, 1H, $J_{4,4a}$ = 7.1 Hz, H-4), 7.03 (t, 1H, *J* = 7.4 Hz, H-7 or H-8), 7.13–7.19 (overlapped peaks, 3H, H-8 or H-7, H-6, H-9),7.25–7.31 (overlapped peaks, 5H, 4-C_6H_5), 7.56, 7.63 (2 x d, 4H, *J* = 8.4 Hz, 1-C_6H_4) ppm. **^{13}C NMR** (150 MHz, $CDCl_3$) δ 32.4; 42.1; 48.4; 122.2; 122.9; 124.0; 125.6; 126.9; 128.2; 128.7; 129.2; 129.3; 130.3; 131.9; 132.2; 133.7; 135.6; 142.0; 146.4; 168.5 ppm. **UV-Vis** (CH_2Cl_2) λ_{max} (log ε) [nm]: 282 (3.77), 295 (sh, 3.82), 317 (3.92); (acetonitrile) λ_{max} (log ε) [nm]: 280 (3.80), 295 (sh, 3.83), 313 (3.90). **HRMS** (APCI+): calcd. for $C_{24}H_{18}BrO_2$ $[M+H]^+$: 417.0485; found: 417.0467; calcd. for $C_{23}H_{16}Br$ $[M-COOH]^+$: 371.0430; found: 371.0424.

(4*R*,4a*S*)-1-(4-Bromophenyl)-4-phenyl-4,4a-dihydroindeno[2,1-*c*]pyran-3(9*H*)-one (3''b)

White solid; yield 4%; mp. 201–202°C; R_f = 0.3 (pentane/methyl *tert*-butyl ether = 8/1). **^{1}H NMR** (600 MHz, $CDCl_3$) δ 3.75 (d, 1H, $J_{9,9}$ = 20.6 Hz, 1H at position 9), 4.12 (d, 1H, $J_{9,9}$= 20.6 Hz, 1H at position 9), 4.56 (d, 1H, $J_{4a,4}$ = 6.7 Hz, H-4a), 4.87 (d, 1H, $J_{4,4a}$ = 6.7 Hz, H-4), 6.96–7.06 (overlapped peaks, 5H, 4-C_6H_5), 7.14–7.24 (overlapped peaks, 4H, H-5, H-6, H-7, H-8), 7.56, 7.61 (2 x d, 4H, J = 8.6 Hz, 1-C_6H_4) ppm. **^{13}C NMR** (150 MHz, $CDCl_3$) δ 36.2; 48.0; 48.6; 119.0; 123.1; 125.0; 125.2; 127.2; 127.8; 127.9; 128.1; 128.6; 128.7; 131.6; 131.9; 133.3; 138.1; 141.7; 143.1; 169.2 ppm. **UV-Vis** (CH_2Cl_2) λ_{max} (log ε) [nm]: 275 (4.05), 282 (4.06); (acetonitrile) λ_{max} (log ε) [nm]: 275 (3.97), 282 (3.98). **HRMS** (APCI+): calcd. for $C_{24}H_{18}BrO_2$ $[M+H]^+$: 417.0485; found: 417.0471; calcd. for $C_{23}H_{16}Br$ $[M-COOH]^+$: 371.0430; found: 371.0427.

(4*S*,4a*R*)-1-(4-(*tert*-Butyl)phenyl)-4-phenyl-4a,5-dihydroindeno[1,2-*c*]pyran-3(4*H*)-one (2'c)

Chemical Formula: $C_{28}H_{26}O_2$; Molecular Weight: 394.50. White solid; yield 20%; mp. 235-236°C; R_f = 0.70 (pentane/methyl *tert*-butyl ether = 8/1). **^{1}H NMR** (600 MHz, $CDCl_3$) δ 1.38 (s, 9H, $C(CH_3)_3$), 2.76 (dd, 1H, $J_{5\alpha, 5\beta}$ = 16.1 Hz, $J_{5\alpha, 4a}$ = 7.7 Hz, H-5*α*), 2.99 (dd, 1H, $J_{5\beta, 5\alpha}$ = 16.1 Hz, $J_{5\beta, 4a}$ = 8.3 Hz, H-5*β*), 3.69 (m from overlapped ddd, 1H, H-4a), 3.77 (d, 1H, $J_{4,4a}$ = 14.2 Hz, H-4), 7.06 (pseuso t from dd, 1H, J = 7.4 Hz, H-7 or H-8), 7.15–7.19 (overlapped peaks, 2H, *p*-H (4-C_6H_5), H-8 or H-7), 7.23 (d, 1H, J = 7.9 Hz, H-9 or H-6), 7.33 (d, 2H, J = 7.3 Hz, *o*-H (4-C_6H_5)), 7.37 (t, 1H, J = 7.4 Hz, H-6 or H-9), 7.44 (m, 2H, *m*-H (4-C_6H_5)), 7.47, 7.61 (2 x d, 4H, J = 8.3 Hz, 1-C_6H_4) ppm. **^{13}C NMR** (150 MHz, $CDCl_3$) δ 31.4; 35.0; 37.0; 44.1; 52.4; 123.0; 123.7; 125.2; 125.6; 126.9; 128.0; 128.3; 128.4; 129.0; 129.1; 129.9; 136.0; 136.7; 143.8; 145.6; 153.0; 169.7 ppm. **UV-Vis** (CH_2Cl_2) λ_{max} (log ε) [nm]: 282 (3.90), 294 (sh, 3.91), 312 (3.97); (acetonitrile) λ_{max} (log ε) [nm]: 278 (3.83), 308 (3.87). **HRMS** (APCI+): calcd. for $C_{28}H_{27}O_2$ $[M+H]^+$: 395.2006; found: 395.2008; calcd. for $C_{27}H_{25}$ $[M-COOH]^+$: 349.1951; found: 349.1956.

(4*R*,4a*R*)-1-(4-(*tert*-Butyl)phenyl)-4-phenyl-4a,5-dihydroindeno[1,2-*c*]pyran-3(4*H*)-one (2''c)

Chemical Formula: $C_{28}H_{26}O_2$; Molecular Weight: 394.50. White solid; yield 20%; mp. 74–76°C; R_f = 0.4 (pentane/methyl *tert*-butyl ether = 8/1). **^{1}H NMR** (400 MHz, $CDCl_3$) δ 1.38 (s, 9H, $C(CH_3)_3$), 2.79 (dd, 1H, $J_{5\alpha,5\beta}$ = 16.0 Hz, $J_{5\alpha,4a}$ = 8.1 Hz, H-5*α*), 3.06 (dd, 1H, $J_{5\beta,5\alpha}$ = 16.0 Hz, $J_{5\beta,4a}$ = 9.0 Hz, H-5*β*), 3.90 (m from overlapped ddd, 1H, H-4a), 4.28 (d, 1H, $J_{4,4a}$ = 7.1 Hz, H-4), 7.02 (t, 1H, *J* = 7.4 Hz, H-7 or H-8), 7.11–7.18 (overlapped peaks, 2H, H-6 or H-9, H-8 or H-7), 7.21 (d, 1H, *J* = 7.8 Hz, H-9 or H-6), 7.24–7.29 (overlapped peaks, 5H, 4-C_6H_5), 7.49, 7.61 (2 x d, 4H, *J* = 8.4 Hz, 1-C_6H_4) ppm. **^{13}C NMR** (100 MHz, $CDCl_3$) δ 31.4; 32.4; 35.1; 42.0; 48.5; 121.1; 123.0; 125.4; 125.8; 126.7; 128.1; 128.3; 128.4; 129.2; 129.5; 130.0; 134.0; 136.2; 143.4; 146.2; 153.1; 168.9 ppm. **UV-Vis** (CH_2Cl_2) λ_{max} (log ε) [nm]: 280 (3.79), 294 (sh, 3.80), 314 (3.86); (acetonitrile) λ_{max} (log ε) [nm]: 280 (3.70), 312 (3.74). **HRMS** (APCI+): calcd. for $C_{28}H_{27}O_2$ $[M+H]^+$: 395.2006; found: 395.2014.

(4*S*,4a*S*)-1-(4-(*tert*-Butyl)phenyl)-4-phenyl-4,4a-dihydroindeno[2,1-*c*]pyran-3(9*H*)-one (3'c)

Chemical Formula: $C_{28}H_{26}O_2$; Molecular Weight: 394.50. White solid; yield 4%; R_f = 0.5 (pentane/methyl *tert*-butyl ether = 8/1), mixture with **2'c** in a ratio 2/1. **^{1}H NMR** (600 MHz, $CDCl_3$) δ 1.39 (s, 9H, $C(CH_3)_3$), 3.71 (d, 1H, $J_{4a,4}$ = 13.8 Hz, H-4a), 3.98 (d, 1H, $J_{9,9}$ = 20.2 Hz, 1H at position 9), 4.16 (d, 1H, $J_{9,9}$ = 20.2 Hz, 1H at position 9), 4.68 (d, 1H, $J_{4,4a}$ = 13.8 Hz, H-4), 6.06 (d, 1H, *J* = 7.8 Hz, H-5 or H-8), 6.93 (t, 1H, *J* = 7.5 Hz, H-6 or H-7), 7.16–7.22 (overlapped peaks, 2H, H-5 or H-8, H-6 or H-7), 7.34 (m, 2H, *o*-H (4-C_6H_5), signals overlapped with the signals of isomer **2'c**), 7.41–7.51 (overlapped peaks, 5H, *m*-H, *p*-H (4-C_6H_5), *m'*-H (1-C_6H_4)), 7.60 (d, 2H, *J* = 8.4 Hz, *o'*-H (1-C_6H_4)) ppm. **^{13}C NMR** (150 MHz, $CDCl_3$) δ 31.4; 32.4; 35.1; 42.0; 48.5; 121.1; 123.0; 125.4; 125.8; 126.7; 128.1; 128.3; 128.4; 129.2; 129.5; 130.0; 134.0; 136.2; 143.4; 146.2; 153.1; 168.9 ppm. **HRMS** (APCI+): calcd. for $C_{28}H_{27}O_2$ $[M+H]^+$: 395.2006; found: 395.2011.

(4*S*,4a*R*)-1-(3-Methoxyphenyl)-4-phenyl-4a,5-dihydroindeno[1,2-*c*]pyran-3(4*H*)-one (2'd)

Chemical Formula: $C_{25}H_{20}O_3$; Molecular Weight: 368.42. White solid; yield 19%; mp. 144–145°C; R_f = 0.60 (pentane/methyl *tert*-butyl ether = 5/1). **^{1}H NMR** (600 MHz, $CDCl_3$) δ 2.77 (dd, 1H, $J_{5\alpha,5\beta}$ = 16.0 Hz, $J_{5\alpha,4a}$ = 8.0 Hz, H-5*α*), 3.01 (dd, 1H, $J_{5\beta,5\alpha}$ = 16.0 Hz, $J_{5\beta,4a}$ = 9.0 Hz, H-5*β*), 3.68 (m from overlapped ddd, 1H, H-4a), 3.84 (s, 3H, OCH_3), 4.30 (d, 1H, $J_{4,4a}$ = 14.1 Hz, H-4), 7.01 (m, 1H, H-4'), 7.05 (m, 1H, H-6'), 7.15–7.20 (overlapped peaks, 4H, H-6, H-7, H-8, H-9), 7.27 ($s_{(br)}$, 1H, H-2'), 7.33–7.39 (overlapped peaks, 4H, H-5', *p*-H (4-C_6H_5), *m*-H (4-C_6H_5)), 7.45 (m, 2H, *o*-H (4-C_6H_5)) ppm. **^{13}C NMR** (150 MHz, $CDCl_3$) δ 37.0; 44.0; 52.3; 55.5; 113.6; 116.1; 121.2; 123.1; 124.2; 125.2; 127.0; 128.1; 128.5; 129.0; 129.1; 129.8; 143.4; 145.7; 159.8; 169.6 ppm. **UV-Vis** (CH_2Cl_2) λ_{max} (log ε) [nm]: 280 (3.84), 309 (4.87); (acetonitrile) λ_{max} (log ε) [nm]: 276 (3.88), 307 (3.86). **HRMS** (APCI+): calcd. for $C_{25}H_{21}O_3$ $[M+H]^+$: 369.1485; found: 369.1487.

(4*S*,4a*R*)-1-(3-Methoxyphenyl)-4-phenyl-4a,5-dihydroindeno[1,2-*c*]pyran-3(4*H*)-one (2''d)

Chemical Formula: $C_{25}H_{20}O_3$; Molecular Weight: 368.42. White solid; yield 16%; mp. 62–63°C; R_f = 0.4 (pentane/methyl *tert*-butyl ether = 3/2). **^{1}H NMR** (600 MHz, $CDCl_3$) δ 2.80 (dd, 1H, $J_{5\alpha,5\beta}$ = 16.0 Hz, $J_{5\alpha,4a}$ = 8.0 Hz, H-5*α*), 3.06 (dd, 1H, $J_{5\beta,5\alpha}$ = 16.0 Hz, $J_{5\beta,4a}$ = 9.0 Hz, H-5*β*), 3.84 (s, 3H, OC*H*$_3$), 3.90 (m from overlapped ddd, 1H, H-4a), 4.30 (d, 1H, $J_{4,4a}$ = 7.1 Hz, H-4), 7.00–7.03 (overlapped peaks, 2H, H-4', H-6'), 7.11–7.19 (overlapped peaks, 4H, H-6, H-7, H-8, H-9), 7.26–7.30 (overlapped peaks, 6H, H-2', H-5', *o*-H (4-C_6H_5), *m*-H (4-C_6H_5)), 7.39 (m, 1H, *p*-H (4-C_6H_5)) ppm. **^{13}C NMR** (150 MHz, $CDCl_3$) δ 32.2, 41.8, 48.3, 55.4, 113.6, 115.8, 120.9, 121.5, 123.0, 125.3, 126.6, 128.0, 128.4, 129.0, 129.3, 129.9, 133.7, 134.0, 135.7, 142.8, 146.1, 159.9, 168.7 ppm. **UV-Vis** (CH_2Cl_2) λ_{max} (log ε) [nm]: 280 (3.71), 311 (3.74); (acetonitrile) λ_{max} (log ε) [nm]: 276 (3.70), 309 (3.68). **HRMS** (APCI+): calcd. for $C_{25}H_{20}O_3$ $[M]^+$: 368.1407; found: 368.1400; calcd. for $C_{24}H_{19}O$ [M-COOH]+: 323.1430, found: 323.1432.

General procedure for the synthesis of indenopyrones 4–6:

DDQ (3.6 mmol) was added to a solution containing a mixture of isomers **2** and **3** (3 mmol) in anhydrous toluene (80 ml) and the reaction mixture was heated at 80 °C in an inert atmosphere for 6 days. After being cooled to room temperature the solution was filtered and the filtrate was washed several times with water and brine. The organic phase was dried over $MgSO_4$ and concentrated to dryness. The crude product was purified by column chromatography on silica gel yielding the desired compounds.

1,4-Diphenylindeno[2,1-*c*]pyran-3,9-dione (6a)

Chemical Formula: $C_{24}H_{14}O_3$; Molecular Weight: 350.37. Yellow solid; yield 15%; mp. 281-282°C; R_f = 0.2 (petroleum ether/diethyl ether = 7/1). **^{1}H NMR** (600 MHz, $CDCl_3$) δ 6.87 (d, 1H, *J* = 7.8 Hz, H-5), 7.38 (t, 1H, *J* = 7.5 Hz, H-6), 7.49–7.62 (overlapped peaks, 9H, H-7, 4-C_6H_5, *m',p'*-H (1-C_6H_5), 7.87 (d, 1H, *J* = 7.8 Hz, H-8), 8.18 (d, 2H, *J* = 8.4 Hz, *o'*-H (1-C_6H_5)) ppm. **^{13}C NMR** (150 MHz, $CDCl_3$) δ 112.6; 120.1; 124.5; 125.9; 128.4; 129.4; 129.5; 129.7; 129.8; 129.9; 132.3; 132.6; 132.8; 134.6; 139.0; 139.6; 149.2; 161.6; 162.3; 186.6 ppm. **UV-Vis** (CH_2Cl_2) λ_{max} (log ε) [nm]: 275 (3.96), 343 (3.74). **HRMS** (APCI+): calcd. for $C_{24}H_{15}O_3$ $[M+H]^+$: 351.1016; found: 351.1024.

1-(4-(*tert*-Butyl)phenyl)-4-phenylindeno[2,1-*c*]pyran-3(9*H*)-one (5c)

Chemical Formula: $C_{28}H_{24}O_2$; Molecular Weight: 392.49. Yellow solid; yield 17%; mp. 173-174°C; R_f = 0.2 (pentane/ methyl *tert*-butyl ether = 8/1). **^{1}H NMR** (400 MHz, $CDCl_3$) δ 1.38 (s, 9H, $C(CH_3)_3$), 4.19 (s, 2H, H-9), 6.81 (d, 1H, *J* = 7.9 Hz, H-5 or H-8), 7.08 (t, 1H, *J* = 7.4 Hz, H-6 or H-7), 7.39 (t, 1H, *J* = 7.4, H-7 or H-6), 7.46-7.56 (overlapped peaks, 8H, H-5 or H-8, o, m, *p*-H (4-C_6H_5), *m'*-H (1-C_6H_4-*p*-*t*Bu)), 7.92 (d, 2H, *J* = 8.6 Hz, *o'*-H (1-C_6H_4-*p*-*t*Bu)) ppm. **^{13}C NMR** (100 MHz, $CDCl_3$) δ 29.8; 31.3; 35.1; 117.2; 118.3; 125.3; 125.9; 126.2; 127.2; 127.4; 128.7; 129.2; 129.8; 130.0; 131.3; 134.0; 137.1; 146.7; 153.0; 153.6; 154.7; 163.8 ppm. **UV-Vis** (CH_2Cl_2) λ_{max} (log ε) [nm]: 276 (4.32), 313 (3.96), 373 (4.16). **HRMS** (APCI+): calcd. for $C_{28}H_{25}O_2$ $[M+H]^+$: 393.1849; found: 393.1852.

1-(4-(*tert*-Butyl)phenyl)-4-phenylindeno[1,2-*c*]pyran-3(5*H*)-one (4c)

Chemical Formula: $C_{28}H_{24}O_2$; Molecular Weight: 392.49. Yellow solid; yield 5%; mp. 182-184°C; R_f = 0.4 (pentane/ diethyl ether = 4/1). **^{1}H NMR** (600 MHz, $CDCl_3$) δ 1.21 (s, 9H, $C(CH_3)_3$), 3.32 (s, 2H, H-5), 6.86 (m, 1H, H-7 or H-8), 6.92 (m, 1H, H-7 or H-8), 6.98 (m, 1H, H-6 or H-9), 7.18–7.20 (m, 1H, *p*-H (4-C_6H_5)), 7.27–7.31 (overlapped peaks, 4H, *m*-H (4-C_6H_5), *m'*-H (1-C_6H_4-*p*-*t*Bu)), 7.48 (d, 2H, *J* = 7.2 Hz, *o*-H (4-C_6H_5)), 7.57 (d, 1H, *J* = 7.8 Hz, H-6 or H-9), 7.72 (d, 2H, *J* = 8.4 Hz, *o'*-H (1-C_6H_4-*p*-*t*Bu)) ppm. **^{13}C NMR** (150 MHz, $CDCl_3$) δ 31.2; 34.9; 36.8; 120.5; 121.8; 122.1; 125.1; 126.0; 127.1; 128.0; 128.4; 128.5; 129.1; 130.1; 130.5; 134.7; 137.0; 142.0; 153.9; 154.0; 157.9; 161.0 ppm. **UV-Vis** (CH_2Cl_2) λ_{max} (log ε) [nm]: 277 (3.84); 365 (3.56). **HRMS** (APCI+): calcd. for $C_{28}H_{25}O_2$ $[M+H]^+$: 393.1849; found: 393.1844.

Additional Data

1. UV–vis spectra recorded in dichloromethane of **2'a–d**, **2''b–d** and **3''b**

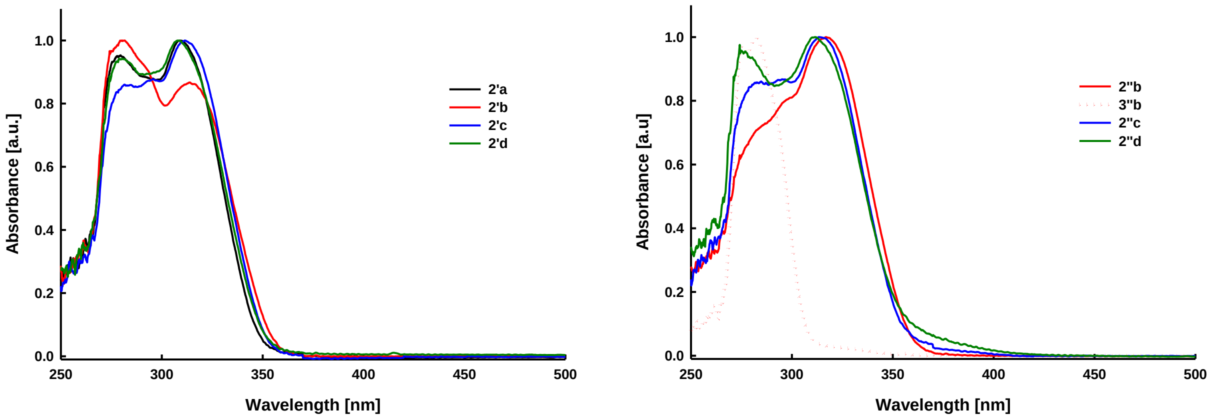

Figure S1: Normalized absorption spectra of dihydroindenopyrones **2'a–d**, **2''b–d** and **3''b** recorded in dichloromethane (298 K).

2. Fluorescence in solution (acetonitrile): spectra of dihydroindenopyrones **2''b–d** and **3''b** (Figure 2) and calculation of quantum yields

The quantum yields in acetonitrile were calculated using 9,10-diphenylanthracene (Φ = 0.90) as a standard material according to eq. (1) [1].

$$\Phi_F = \Phi_{ref} \left(\frac{S\,sample}{S\,ref}\right)\left(\frac{A\,ref}{A\,sample}\right)\left(\frac{n^2 sample}{n^2 ref}\right) \quad (1)$$

where Φ_F is the quantum yield of samples, Φ_{ref} is the quantum yield of 9,10-diphenylanthracene, A_{ref}, S_{ref}, n_{ref} and A_{sample}, S_{sample}, n_{sample} are the absorbance at the excited wavelengths, the integrated emission band area and the solvent refractive index of the standard and the sample, respectively.

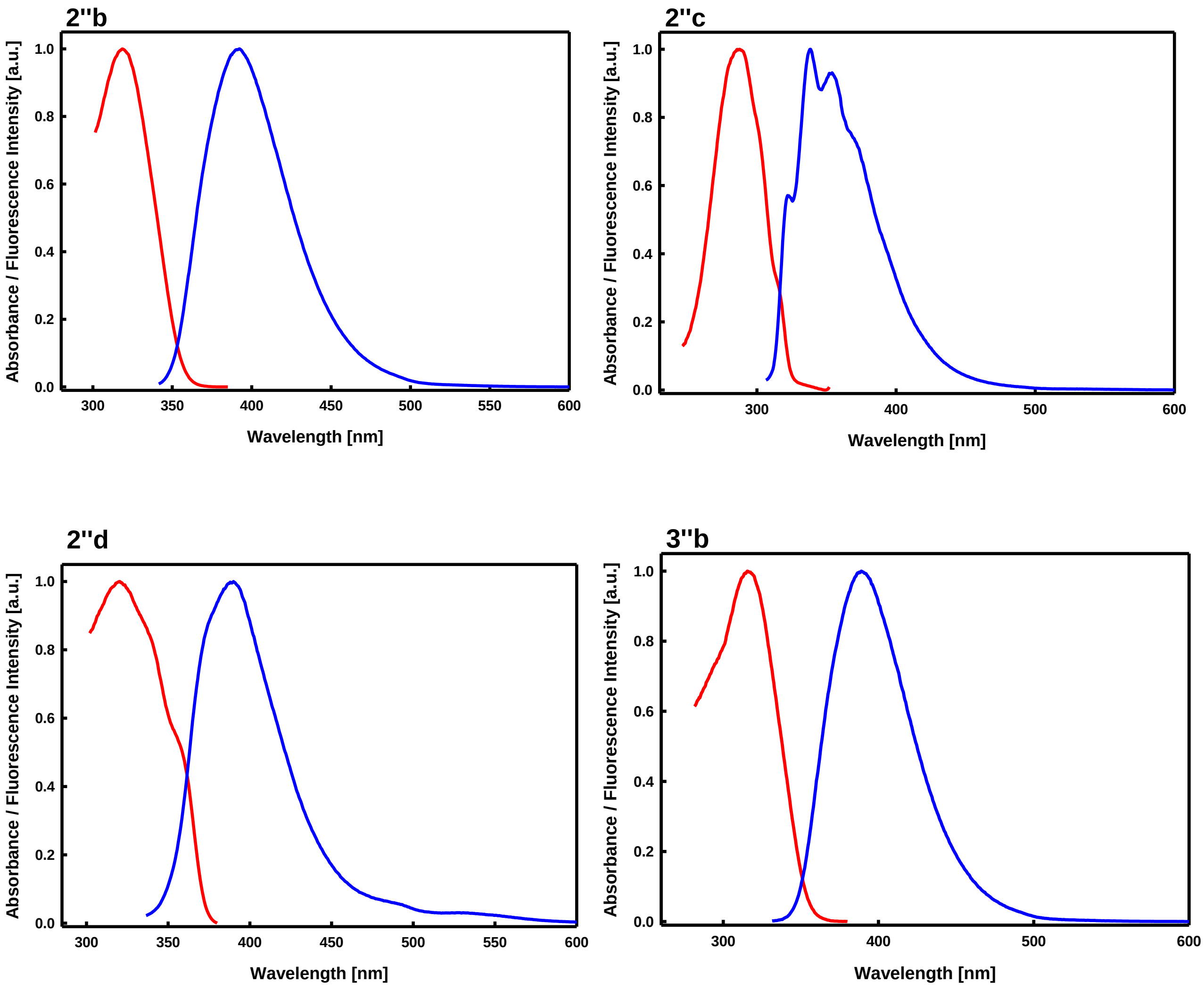

Figure S2: Normalized UV–vis (red) at excitation wavelengths and fluorescence (blue) spectra of dihydroindenopyrones **2''b–d** and **3''b**.

3. ^{1}H NMR spectra of compounds **2'a**, **3''a**, **2'c**, **2''c**, **2'd**, **2''d** (Figure S3)

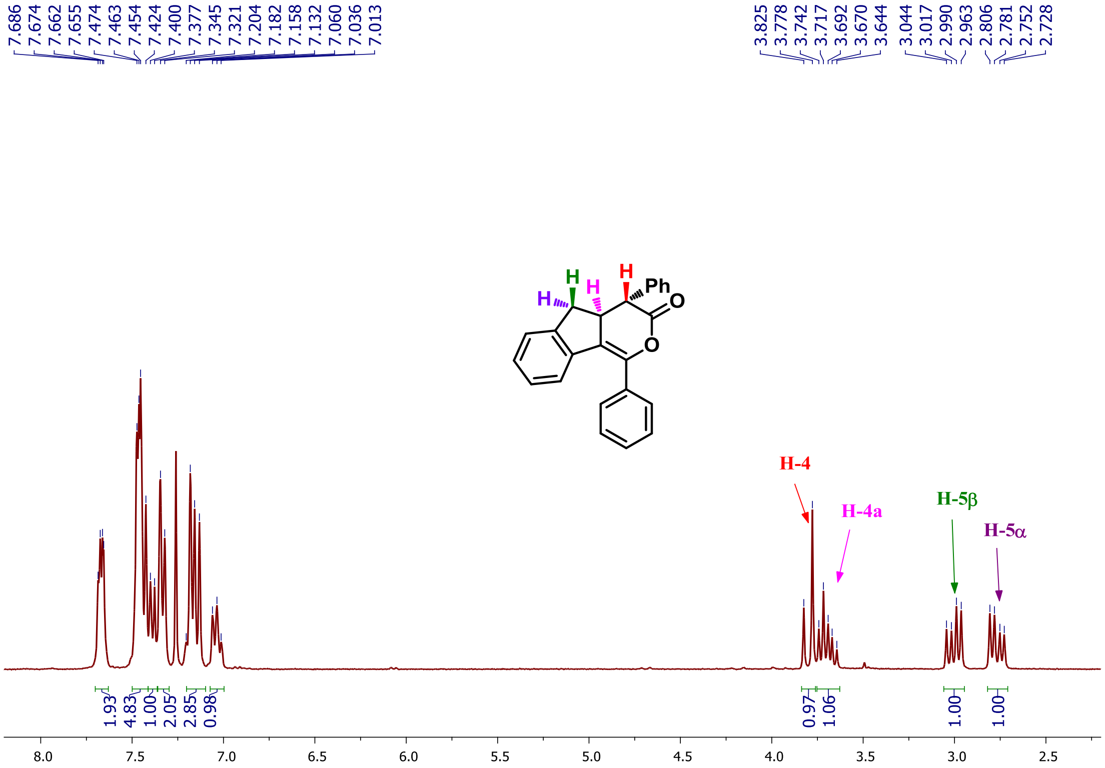

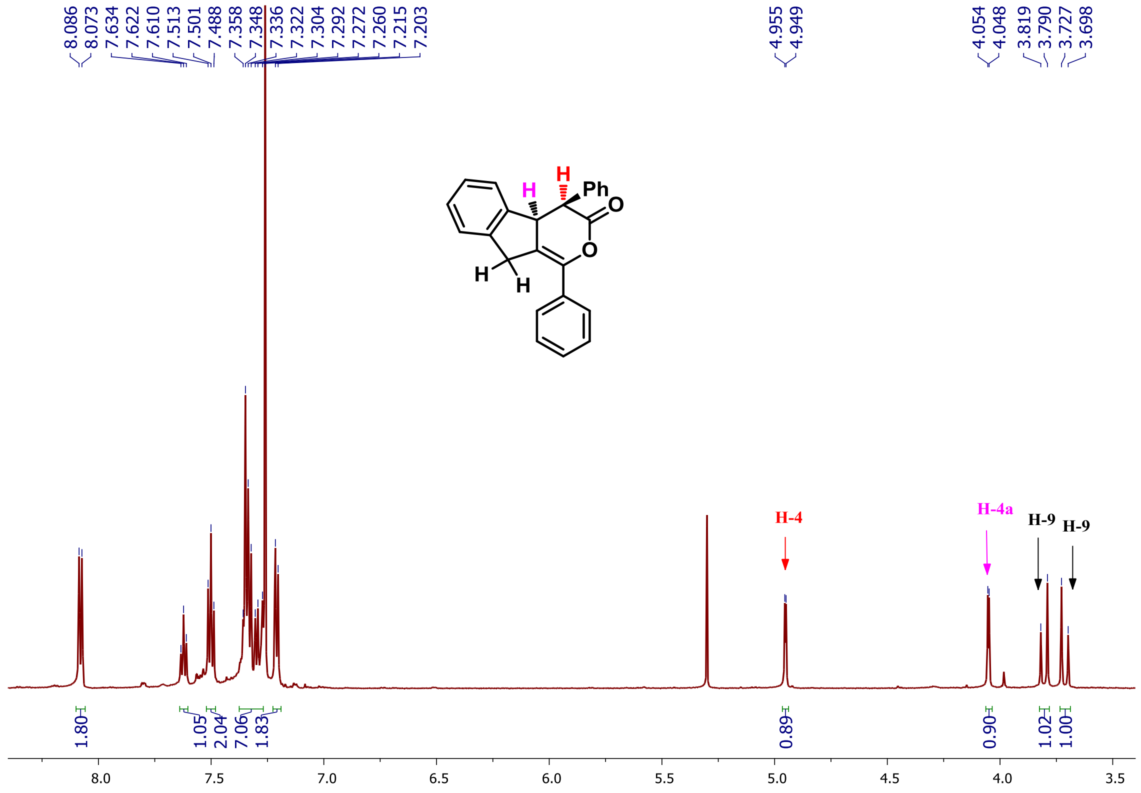

H-4
H-4a
H-9
H-9

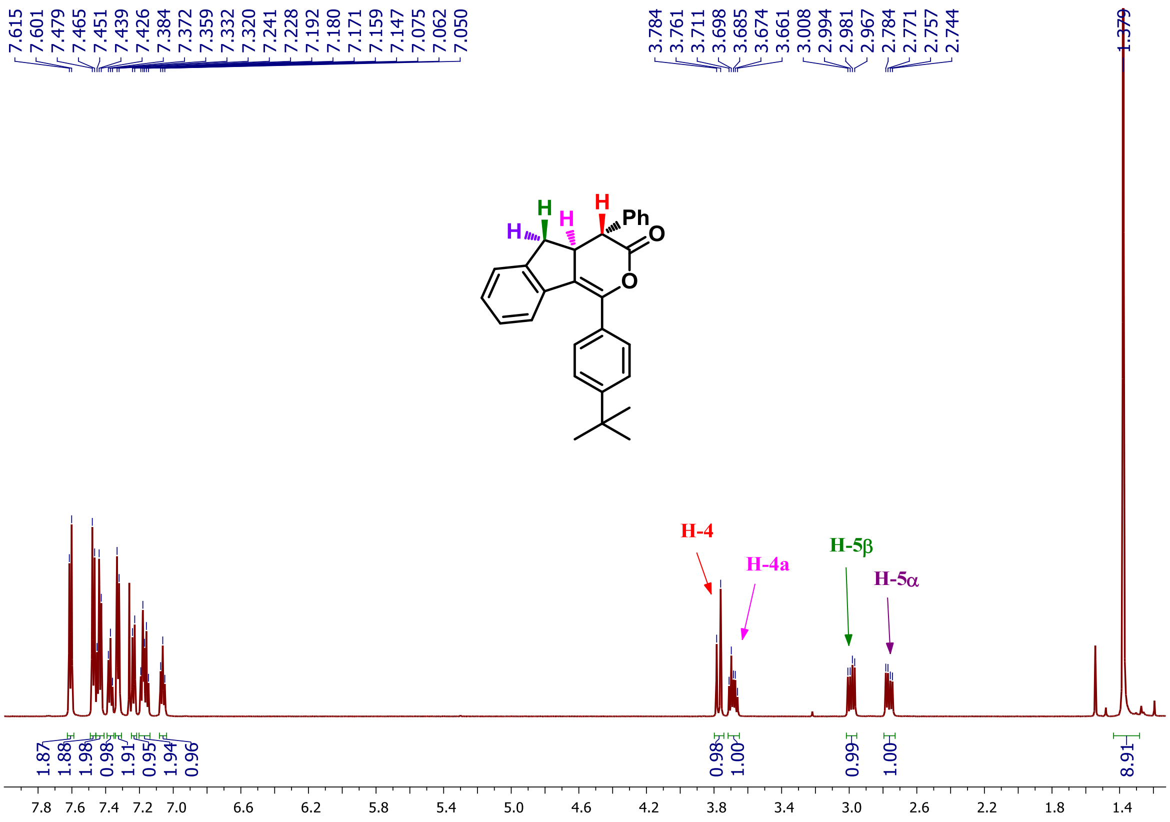

H-4
H-4a
H-5β
H-5α

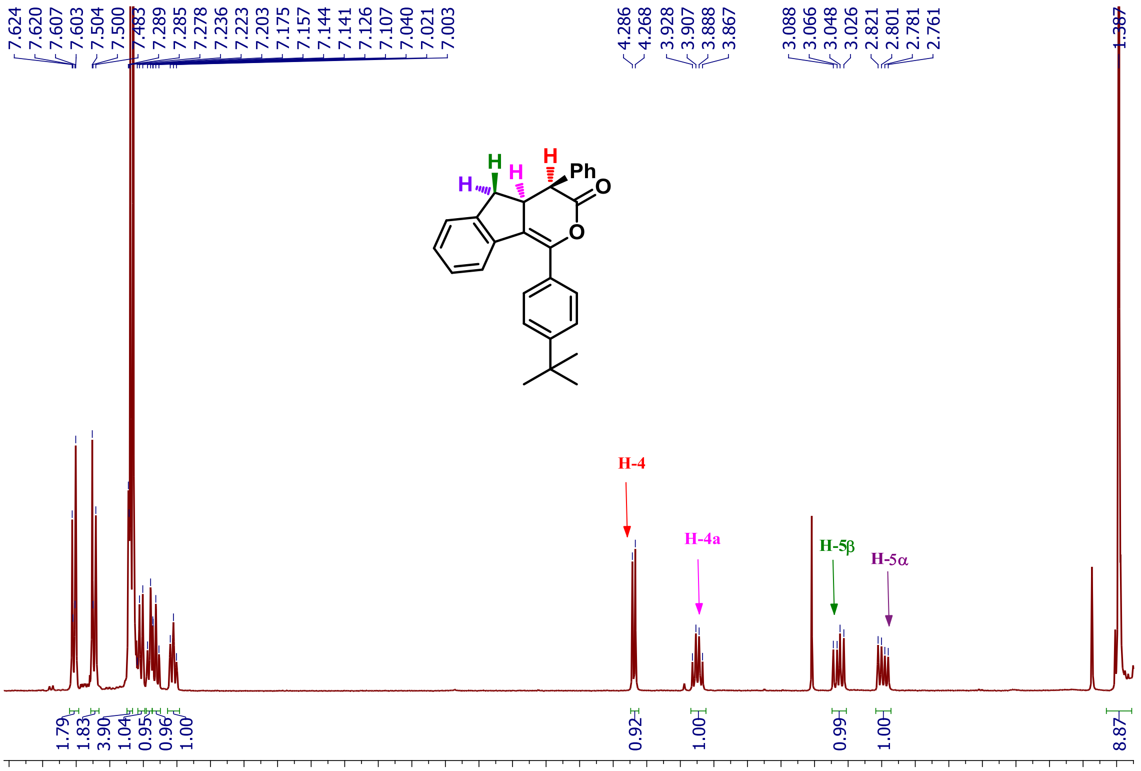

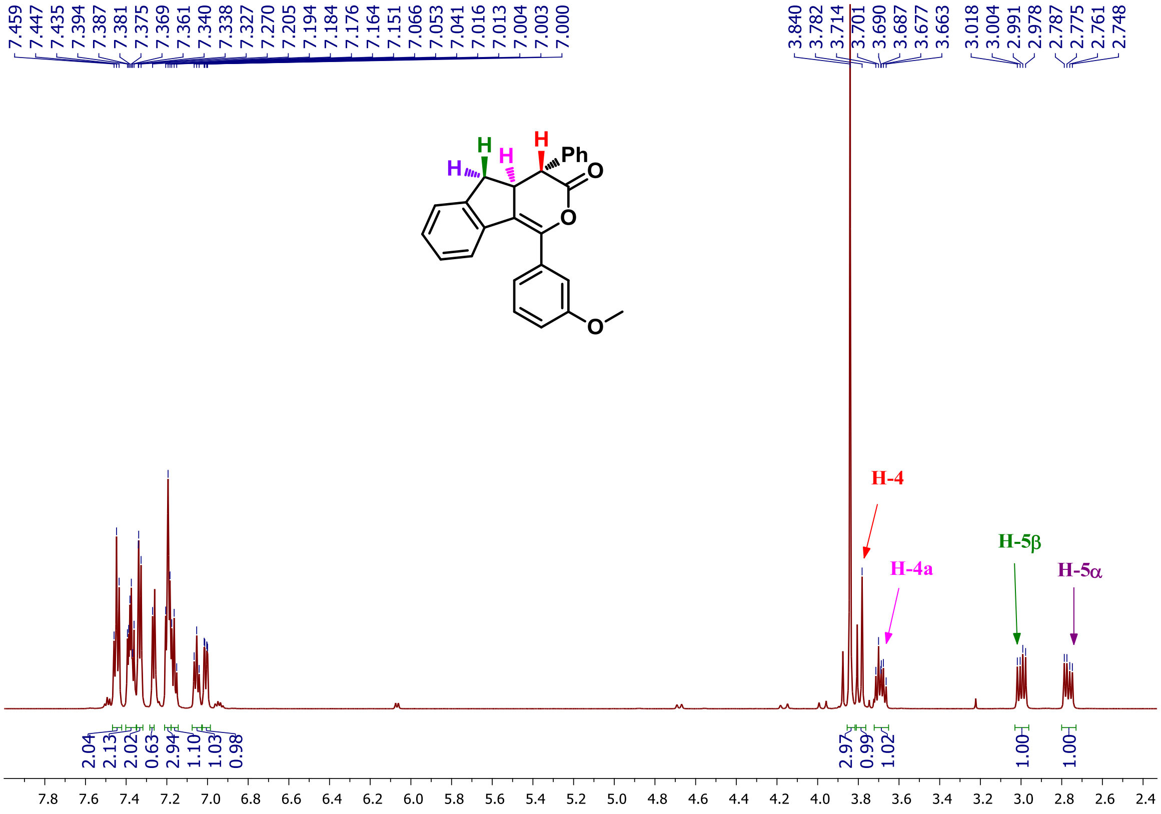

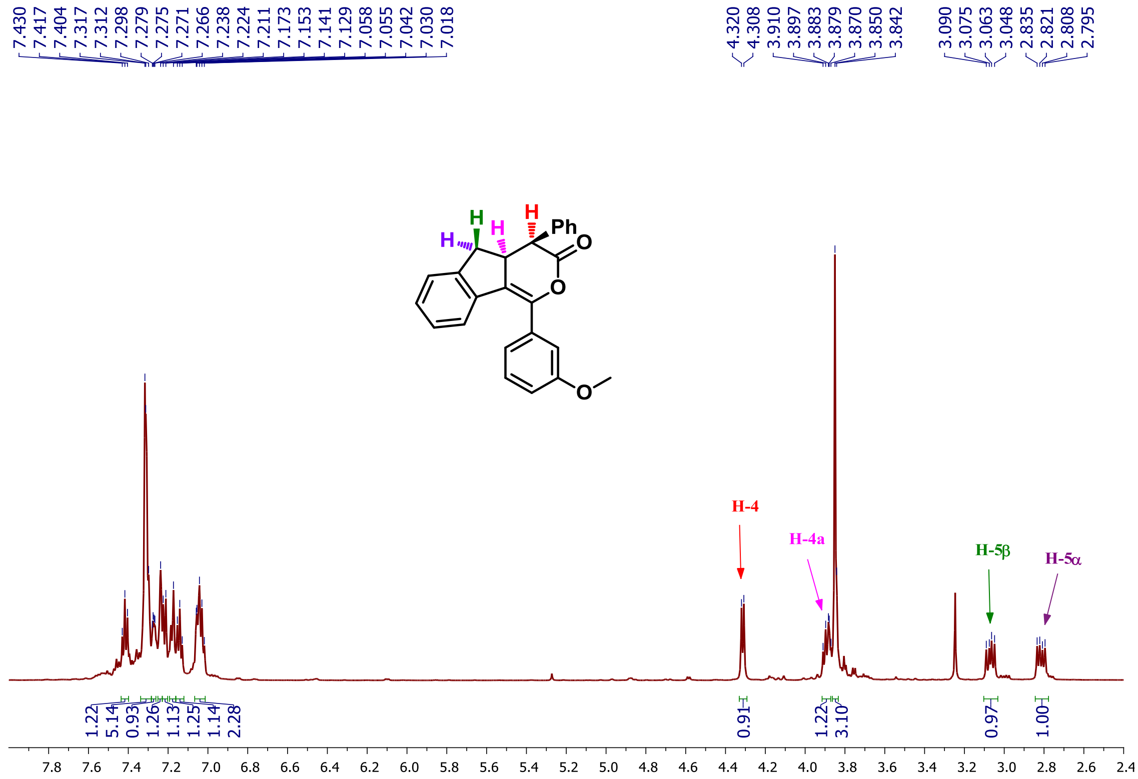
7.430
7.417
7.404
7.317
7.312
7.298
7.279
7.275
7.271
7.266
7.238
7.224
7.211
7.173
7.153
7.141
7.129
7.058
7.055
7.042
7.030
7.018
4.320
4.308
3.910
3.897
3.883
3.879
3.870
3.850
3.842
3.090
3.075
3.063
3.048
2.835
2.821
2.808
2.795
H
H
H
H
Ph
O
O
O
H-4
H-4a
H-5β
H-5α
1.22
5.14
0.93
1.26
1.13
1.25
1.14
2.28
0.91
1.22
3.10
0.97
1.00
7.8
7.6
7.4
7.2
7.0
6.8
6.6
6.4
6.2
6.0
5.8
5.6
5.4
5.2
5.0
4.8
4.6
4.4
4.2
4.0
3.8
3.6
3.4
3.2
3.0
2.8
2.6
2.4

4. ^{1}H NMR spectra of compounds **4–6** (Figure S4)

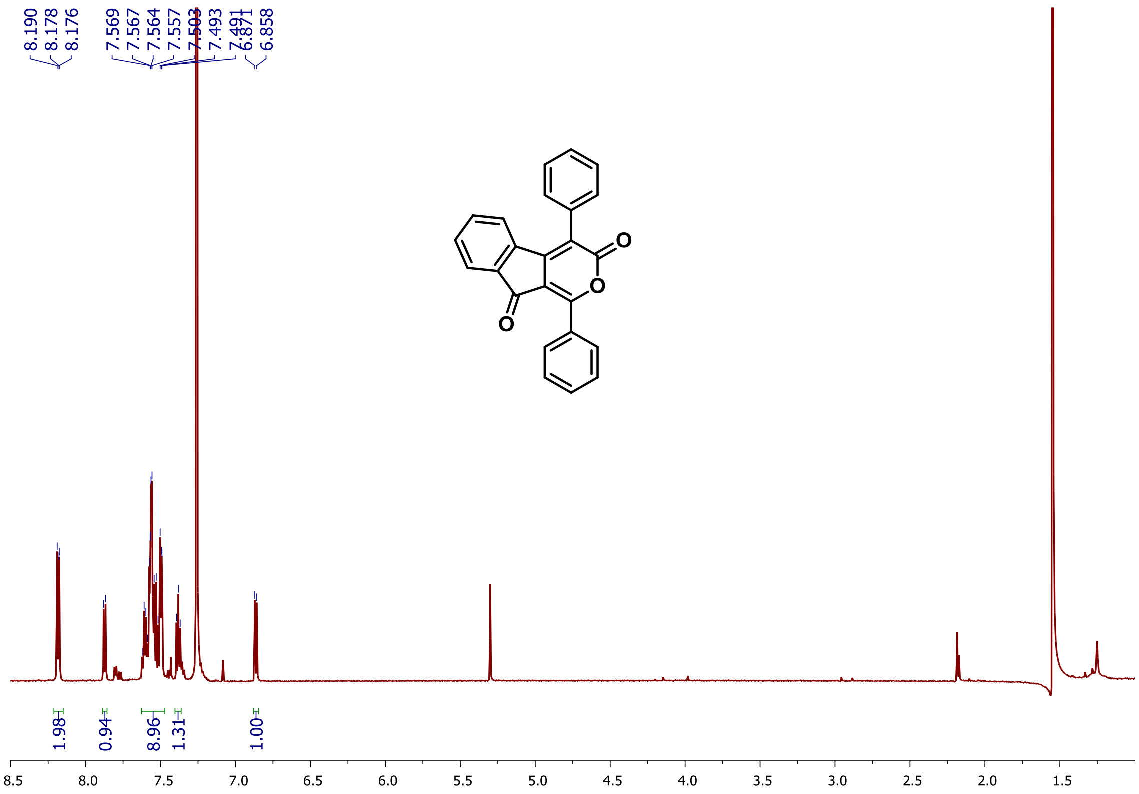

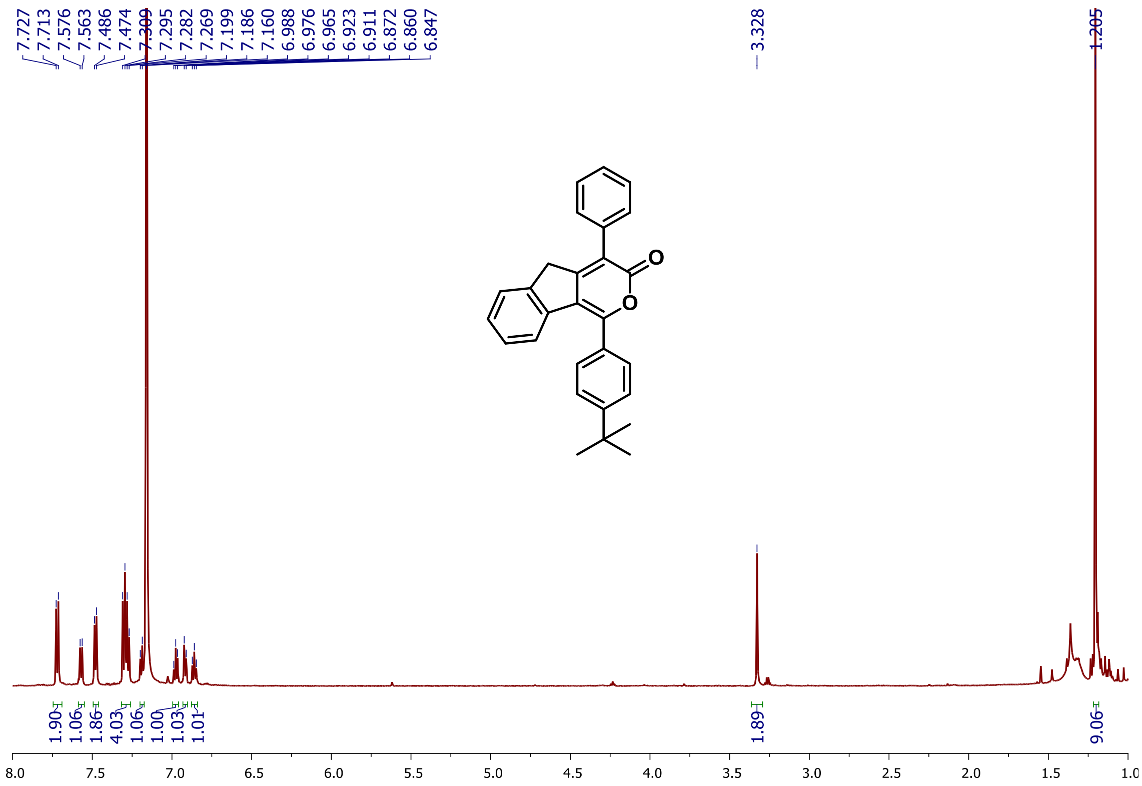

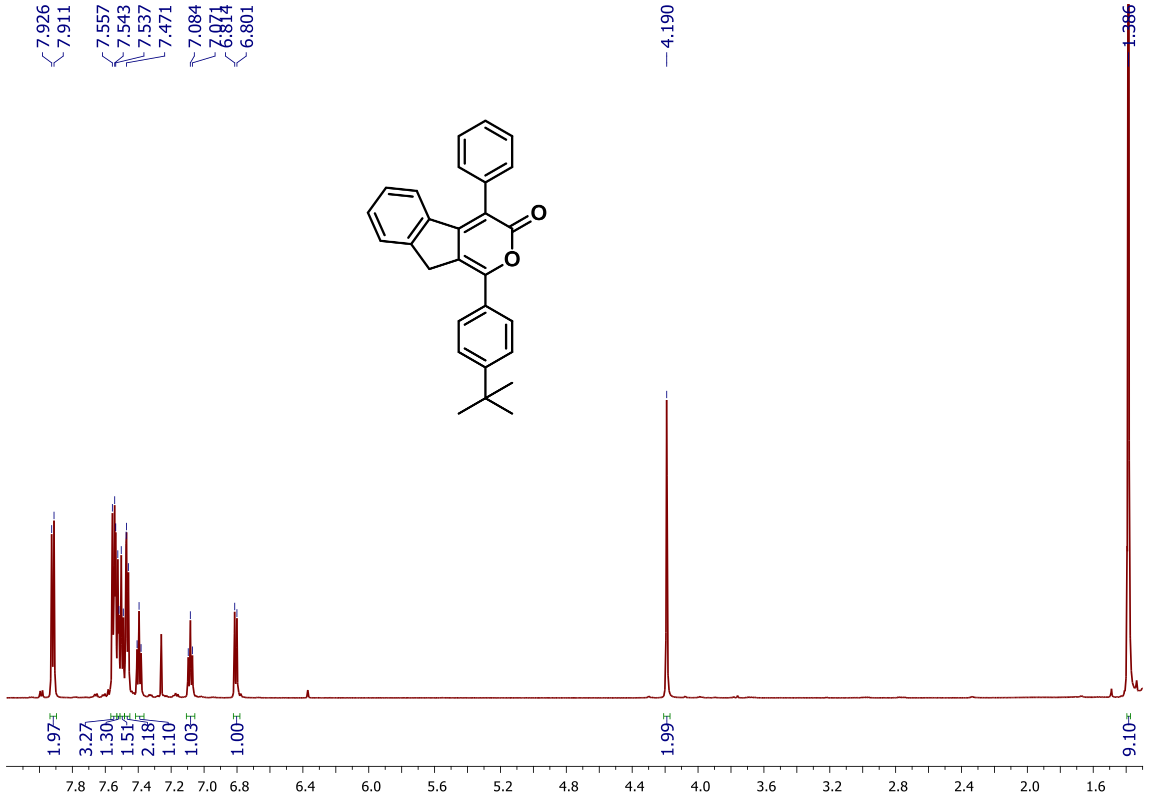

5. Fluorescence of α-pyrones **4c, 5c** and **6a** in solution (dichloromethane)

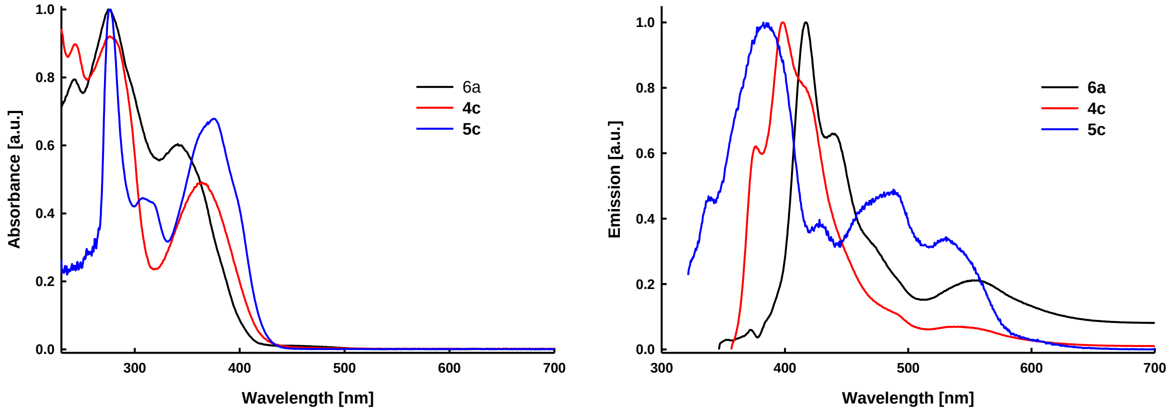

Figure S5: Normalized absorption (left) and fluorescence (right) spectra of **4c, 5c** and **6a** measured in dichloromethane.

6. Crystallographic data of compound **6a**

Single crystals were obtained by slow evaporation of the deuterated chloroform solution of the NMR sample. Data were collected on a Bruker SMART APEX diffractometer, using graphite-monochromated Mo-Kα radiation (λ = 0.71073 Å). For this purpose the crystals were attached with Paratone oil to a cryo-loop. Data was collected at room temperature. The structure was refined with anisotropic thermal parameters. All hydrogen atoms were refined with a riding model and a mutual isotropic thermal parameter. For structure solving and refinement the software package SHELX-97 and SHELXL-2014 were used [2,3]. The drawings were created with the Diamond program [4]. CCDC reference number: 1450552.

Table S1: Hydrogen bond geometry (Å, °).

D*-H····*A	***D*-H**	**H····*A***	***D* ····*A***	***D*-H····*A***
C8-H8····O1^{i}	0.93	2.81	3.629(7)	148
C22-H22····O2^{ii}	0.93	2.65	3.447(8)	144
C16-H16····O3^{iii}	0.93	2.56	3.427(7)	156
C15-H15····O2*	0.93	2.86	3.774(7)	169
C17-H17····O2$^{\#}$	0.93	2.76	3.675(7)	167

Symmetry equivalent atoms are given by: (i) x, -1+y, z; (ii) 1-x, -½+y, ½-z; (iii) -x, ½+y, -½-z; (*) –x, 2-y, -z; and (#) x, $^{3}/_{2}$-y, -½+z.

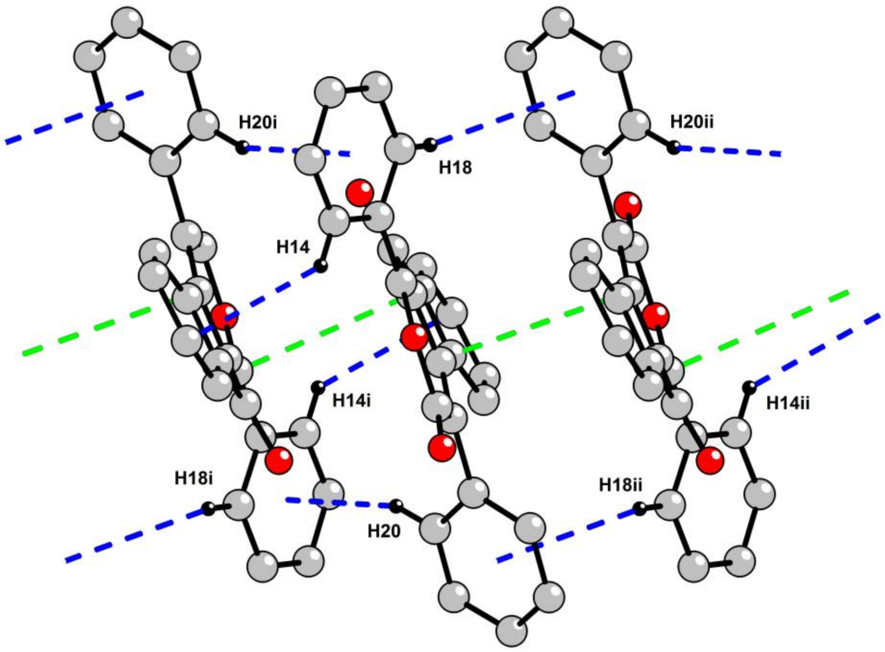

Figure S6: View along the *b* axis in a crystal of **6a**, showing details from the layers connected through weak C–H···π bonds (blue dashed lines) and π···π interactions (green dashed lines). Hydrogen atoms not involved in weak bonds are omitted for clarity. Symmetry equivalent atoms are given by: (i) -x, 1-y,-z; (ii) 1-x, 1-y, -z.

References

1. Shaki, H.; Gharanjig, K.; Rouhani, S.; Khosravi, A. *J. Photo. Photobiol A: Chem.* **2010**, *216*, 44.

2. Sheldrick, G. M. *Acta Crystallogr., Sect. A: Found. Crystallogr.* **2008**, *64*, 112–122.

3. Sheldrick, G. M. *Acta Crystallogr., Sect. C: Cryst. Struct. Commun.* **2015**, *71*, 3–8.

4. *Diamond – Crystal and Molecular Structure Visualisation*, Crystal Impact – Dr. H. Putz and Dr. K. Brandenburg GbR, Kreuzherrenstr. 102, 53227 Bonn, Germany, 2015.
